# Supplementary material for: Mutations in PCYT2 disrupt etherlipid biosynthesis and cause a complex hereditary spastic paraplegia
Source: Brain. 2019 Oct 22;142(11):3382–97. doi: 10.1093/brain/awz291 (PMC6821184; doi:10.1093/brain/awz291)
Supplement: awz291_Supplementary_Data [file awz291_supplementary_data.zip › awz291-Suppl_data/Supplementary_Data4.zip!/Supplementary information 4 - plasma lipidomics.zip!/Plasma lipidomics/Control vs Patient3a/Statistical plots and data/OPLS-DA_scores.pdf]

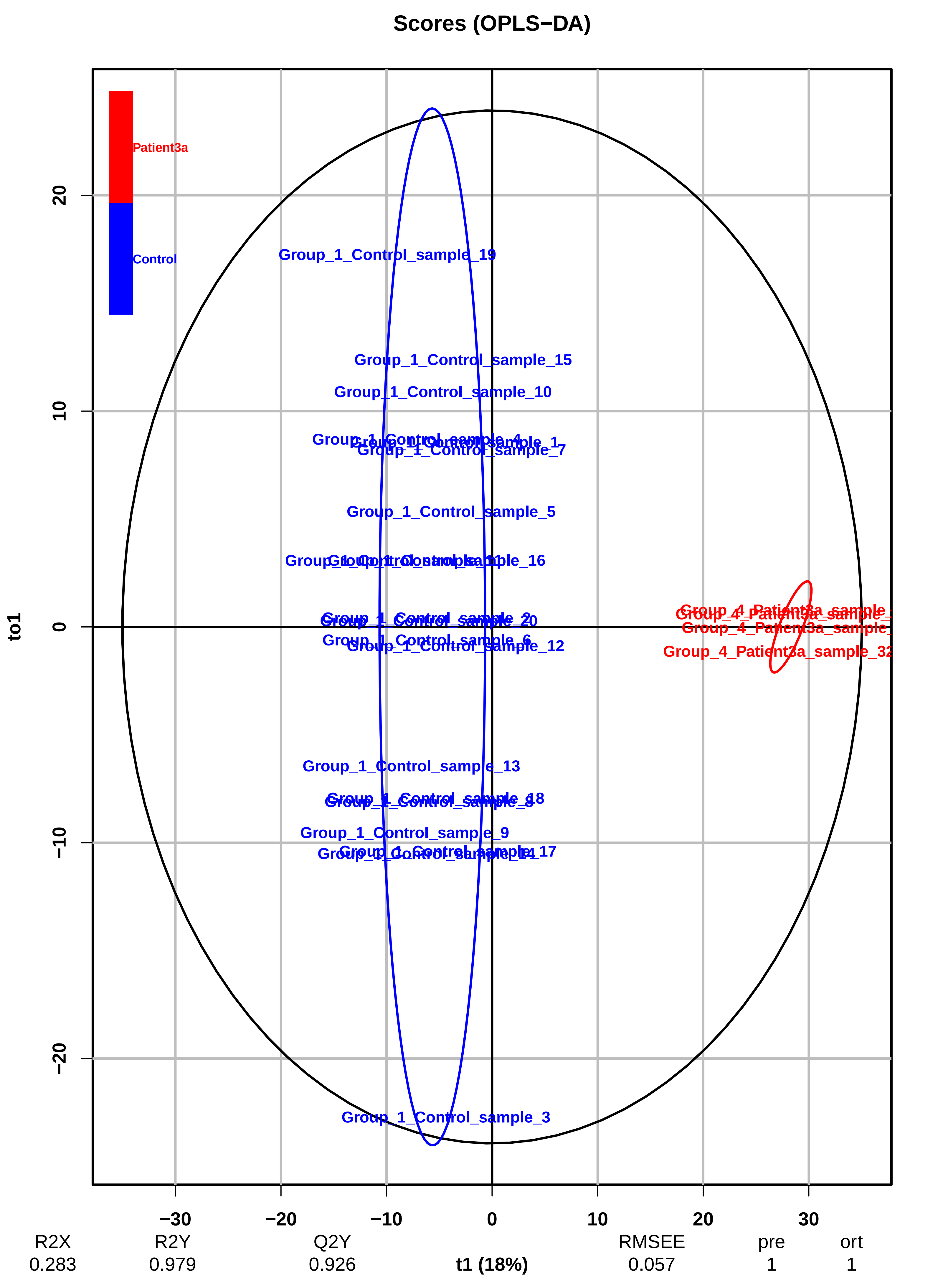
Scores (OPLS−DA)
Patient3a
Control
Group_1_Control_sample_19
Group_1_Control_sample_15
Group_1_Control_sample_10
Group_1_Control_sample_7
Group_1_Control_sample_5
Group_1_Control_sample_12
Group_1_Control_sample_13
Group_1_Control_sample_9
Group_1_Control_sample_3
20
10
0
−10
−20
to1
−30
−20
−10
0
10
20
30
t1 (18%)
R2X
0.283
R2Y
0.979
Q2Y
0.926
RMSEE
0.057
pre
1
ort
1
